# Morphological and immunohistochemical characterisation of seminomas in Norwegian dogs

**DOI:** 10.1186/1751-0147-54-52

**Published:** 2012-09-17

**Authors:** Tor Espen Thorvaldsen, Ane Nødtvedt, Tom Grotmol, Gjermund Gunnes

**Affiliations:** 1 Department of Companion Animal Clinical Sciences, Norwegian School of Veterinary Science, PO Box 8146 Dep, 0033, Oslo, Norway; 2 Institute of Population-Based Cancer Research, Cancer Registry of Norway, PO Box 5313, Majorstuen, 0304, Oslo, Norway; 3 Department of Basic Sciences & Aquatic Medicine, Norwegian School of Veterinary Science, PO Box 8146 Dep, 0033, Oslo, Norway

**Keywords:** Dog, Seminoma, Testis, Tumour

## Abstract

**Background:**

Seminomas in the dog have traditionally been assumed to resemble human spermatocytic seminomas, based on their low malignancy and high occurrence in old individuals. However, recently published studies indicate that canine seminomas can be classified as classical and spermatocytic seminomas in a similar way as in man, and that classical seminomas comprise a substantial proportion of seminomas in the dog. These two factors both contribute to increasing the potential of canine seminoma as a relevant model for human testicular cancer. The aim of the present study was to characterise seminoma in Norwegian dogs using morphology and immunohistochemistry, and determine whether these tumours are comparable with human classical seminoma.

**Methods:**

By applying diagnostic criteria from human pathology, 45 seminomas from the Norwegian Canine Cancer Register were examined histologically with hematoxylin and eosin (HE) and periodic acid-Schiff (PAS) stains. All sections were stained immunohistochemically with antibodies against human placental alkaline phosphatase (PLAP) and the transmembrane receptor c-KIT.

**Results:**

Although two of the seminomas showed immunohistochemical staining characteristics indicative of classical seminoma (PLAP+/c-KIT+), all 45 examined seminomas were morphologically consistent with spermatocytic seminoma.

**Conclusions:**

The value of canine seminoma as a model for SE in man remains unclear. Among the 45 investigated tumours from Norwegian dogs, none were classified as classical seminoma based on morphological criteria consistent with human seminomas. Regional or breed differences in the occurrence of classical seminoma in the dog, as well as the lack of uniform diagnostic criteria, might explain the discrepancy between the findings in the current study and the results presented by other authors.

## Background

The vast majority of human testicular tumours are germ cell neoplasms. These are histologically classified as seminomas and non-seminomas [1]. The WHO classification of testicular tumours in man includes two types of seminoma: classical seminoma (SE) and spermatocytic seminoma (SS) [2]. SE is the most common malignant tumour among young men, with a peak incidence between the ages of 25 and 35 years. Norway has one of the highest incidences of testicular germ cell tumours in the world with 11.7 cases per 100,000 person-years (age-standardised rates (world) 2005–09) [3]. SS, on the other hand, occurs from the sixth decade of life, is less aggressive and only rarely metastasizes [4]. Unknown environmental factors have been hypothesised to be part of the pathogenesis for SE due to the observed differences in occurrence between regions, in concert with an increase in incidence during the past decades [5].

The difference in biological behaviour between SE and SS is related to their various origins: SE originates from the fetal gonocyte stage and SS from the post-pubertal spermatogonial/spermatocytic stage [6]. Gonocytes are recognised histologically by periodic acid-Schiff (PAS) staining and immunohistochemistry [1]. The cells express the membrane-bound glycoprotein placental-like alkaline phosphatase (PLAP), which can be detected immunohistochemically with a commercial monoclonal antibody. PAS staining detects the presence of glycogen in cells. Neoplastic cells in SE express PLAP and are PAS positive, while the majority of SSs, which are derived from more differentiated cells, do not express PLAP and are PAS negative [1,7]. Immunohistochemical detection of c-KIT protein, which is a transmembrane tyrosine kinase receptor, is another method used for confirming the diagnosis of SE in man. Antibodies against c-KIT detect germinal stem cells and germinal cells at early stages of development [8].

Testicular tumours occur more frequently in the dog than in any other species, including man [9-11]. Sertoli cell tumours, seminomas and interstitial cell tumours (Leydig cell tumours) are the most common histological types [12,13]. Because testicular tumours are frequent in the dog and dogs also share the same environment as man, the canine model has been suggested as a means to investigate the effect of environmental factors on human germ cell tumour occurrence [14].

Canine seminomas are classified according to growth pattern [15], and classification into classical and spermatocytic seminoma has not been established in veterinary pathology. Furthermore, canine testicular germ cell tumours generally develop in middle-aged to old dogs [12,14,16]. On the basis of the low malignancy, low metastatic potential and their tendency to develop in old individuals, it has traditionally been assumed that canine seminomas represent the counterpart to human SS [17]. However, this concept was recently challenged by three publications in which canine seminomas were characterised morphologically and by immunohistochemical analysis for PLAP and c-KIT [16,18,19]. Grieco et al. (2007, 2010) and Kim et al. (2010) classified 22/43 (51%) and 8/23 (35%) of canine seminomas as SE, respectively. More recently, Bush et al. (2011) morphologically evaluated and characterised 347 canine seminomas using diagnostic criteria from human pathology. Immunohistochemical analysis for PLAP, c-KIT, DAZ and DMRT-1 expression was performed and the authors concluded that the investigated canine seminomas closely resembled SS [20].

If the canine model is to aid in the detection of environmental risk factors for human SE, the degree to which the tumours compare across the two species needs to be determined. The aim of the present study was therefore to characterise seminoma in Norwegian dogs using morphology and immunohistochemistry, and determine to what extent these tumours are comparable with human classical seminoma.

## Methods

### Study sample

In this cross-sectional study, 46 seminomas were selected from the Norwegian Canine Cancer Register based on computer-generated random numbers. The full dataset consisted of 214 seminomas submitted to the diagnostic pathology service at the Norwegian School of Veterinary Science between 1998 and 2009. The seminomas in this study were re-examined by an experienced pathologist (GG) during 2010 to confirm the histopathological diagnosis. Tumours were derived from intact dogs ranging in age from 6 to 15 years. Twenty different breeds were represented in the material. Twelve dogs were of either mixed or unknown origin.

### Histopathology

Serial sections (3–4 μm) were obtained and stained with HE and PAS. On the basis of their histological patterns, seminomas were classified as intratubular or diffuse, according to the WHO classification system for tumours of domestic animals [15]. Seminomas showing both intratubular and diffuse neoplastic growth were designated as “intratubular/diffuse”.

### Immunohistochemistry

Immunohistochemistry was performed with PLAP and c-KIT antibodies. The PLAP staining was performed with a mouse monoclonal IgG antibody (Product code M7191, Dako AS, DK-2600 Glostrup, Denmark) and sections (3–4 μm) from formalin fixed, paraffin wax embedded tissue were labelled by the avidin-biotin-peroxidase complex (ABC) procedure with the Vectastain® immunoperoxidase kit (Vector Laboratories, Burlingame, CA 94010, USA). The c-KIT staining was performed on similar tissue sections, with a rabbit polyclonal antibody (Product code A4502, Dako AS) and the EnVision®-kit (Dako AS, DK-2600 Glostrup, Denmark). In brief, the staining protocol was as follows (a detailed protocol may be obtained from the corresponding author):

*PLAP:* Antigen retrieval was done by immersion of tissues in a 0.5 M EDTA buffer at pH 8.0 and heating the tissue and buffer to 92 °C for 5 minutes in a microwave oven at 750 watts. Inhibition of endogenous peroxidase was done with H_2_0_2_ in methanol. Blocking of nonspecific binding was done with equine normal serum diluted with bovine serum albumin (BSA)/tris buffered saline (TBS). Primary antibody was diluted 1:25 in TBS and sections were incubated at 4 °C over-night. Sections were then incubated for 30 minutes with biotinylated secondary antibodies, and subsequently incubated with ABC-complex according to kit instructions. Specific signal was developed by incubation in 3-amino-9-ethylcarbazole (AEC) and the sections were counterstained with hematoxylin.

*c-KIT:* Antigen retrieval was done by immersion of tissues in Tris/EDTA buffer at pH 9.1 and heating the tissue and buffer as described for PLAP. Inhibition of endogenous peroxidase was done with H_2_O_2_ in methanol and blocking of nonspecific binding was done with caprine normal serum diluted with BSA/TBS. Primary antibody was diluted 1:50 in BSA/TBS and sections were incubated at room temperature for one hour. The sections were incubated for 30 minutes with polymer/HRP labelled anti-rabbit secondary antibody (EnVision®-kit, Dako AS, DK-2600 Glostrup, Denmark), developed with chromogen solution according to kit instructions and counterstained with hematoxylin.

Specimens from human SEs were included as positive controls in each immunohistochemical assay. Moreover, peritubular myoid cells and smooth muscle cells of vascular walls were regarded as internal positive controls for the PLAP staining. The human SE was also used as a negative control. In the negative controls, the primary antibody was replaced by an isotype control (irrelevant mouse IgG-kappa, Product code X0931, Dako AS) diluted 1:150 for the PLAP staining and BSA/TBS buffer for the c-KIT staining.

All histological slides were examined using a Leica DM RXA Light microscope (Leica Microsystems AG, Wetzlar, Germany), and digital images were captured with a Spot RT Slider digital camera and software (Diagnostic Instruments Inc., Sterling Heights, MI, USA). Digital images with annotations were assembled in Adobe Photoshop Elements 8 Ver. 8.0 (Adobe Systems Inc., San Jose, California, USA).

## Results

### Study sample

One specimen was removed from the study sample due to artefacts after the immunohistochemical procedure. As a result, 45 seminomas were available for further investigation. The age of one dog was unknown. The mean age for the remaining dogs was 10.1 years. There was no obvious breed predilection in the study sample.

### Histopathology

**Figure 1:**
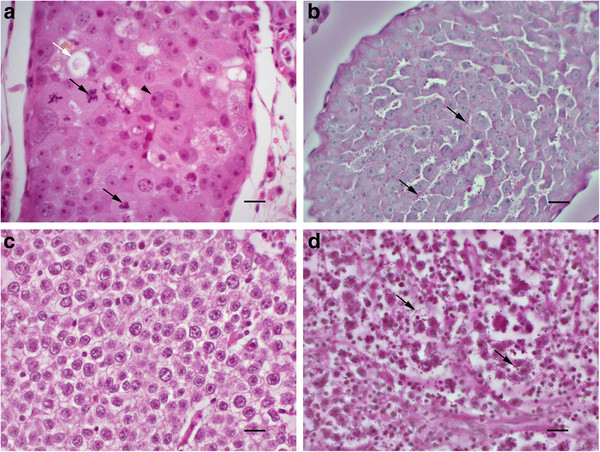
**Histological appearance of canine seminoma observed in the present study compared with human classical seminoma.** (**a**) Canine seminoma with morphology consistent with human spermatocytic seminoma: anisocytosis, anisokaryosis, numerous mitotic figures (black arrows), degenerate cells (white arrow) and multinucleate cells (arrowhead). HE. Bar, 20 μm. (**b**) Canine seminoma (same as in (a)). There are scattered, PAS + granules (arrows) in the cytoplasm of many tumour cells. PAS. Bar, 20 μm. (**c**) Human classical seminoma displaying characteristic monomorphic cells with clear cytoplasm. HE. Bar, 20 μm. (**d**) Human classical seminoma with numerous, PAS + cytoplasmic granules (arrows). PAS. Bar, 20 μm.

All 45 examined seminomas had a morphology consistent with SS, with strong variation in size of tumour cells and tumour nuclei (anisocytosis and anisokaryosis), variable coarseness of nuclear chromatin, often with one or several distinct nucleoli, and a high mitotic index (approximately 1–3 mitotic figures per HPF). The cell borders were moderately distinct, and the cytoplasm was eosinophilic, finely granulated and occasionally vacuolated. There were also scattered, degenerate histiocytes ("starry-sky"-pattern) and multifocal lymphocyte infiltration (Figure 1a). Of these 45 seminomas, 24 were diffuse only, 9 were tubular only, and 12 were both diffuse and tubular. “Starry sky”-pattern and lymphocyte infiltrates were less apparent in intratubular tumour tissue. Five of 45 tumours had some granular, PAS + signal in the cytoplasm of tumour cells (Figure 1b). Four of these cases were intratubular tumours and one was diffuse. The tumour cells in the human seminoma, which was used as a positive control, had a monomorphic appearance, with minimal anisocytosis and anisokaryosis, clear cytoplasm (HE stain) with distinct cell borders, and a low mitotic index (Figure 1c). These cells contained a strong granular, PAS positive cytoplasmic signal (Figure 1d).

### Immunohistochemistry

**Figure 2:**
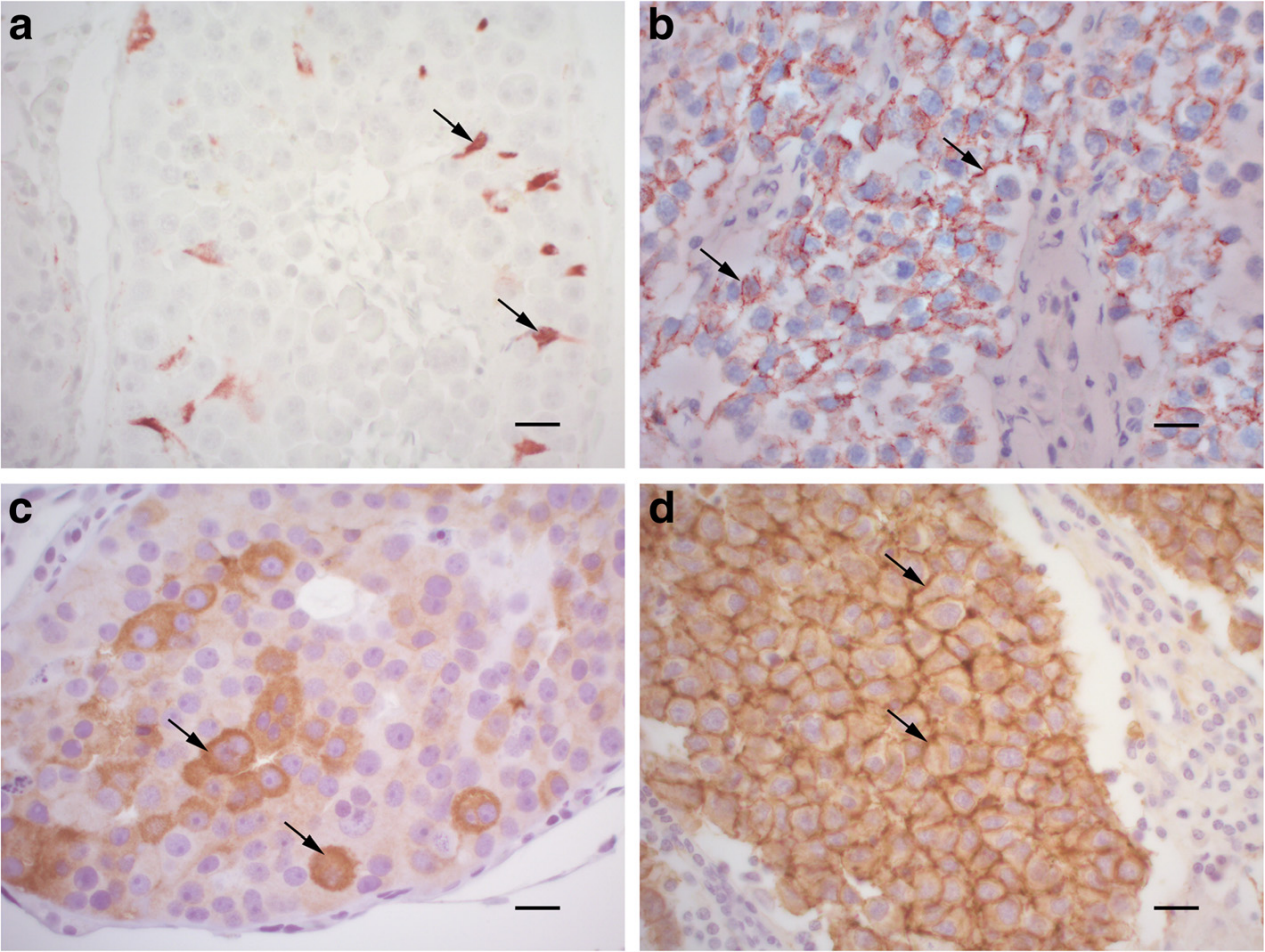
**Immunohistochemistry (IHC) of canine seminoma observed in the present study compared with human classical seminoma.** (**a**) Canine seminoma (same as in Figure 1 a). Scattered cells are strongly positive for PLAP (arrows). PLAP IHC. Bar, 20 μm. (**b**) Human classical seminoma. There is a distinct reticular pattern of PLAP-staining (arrows), indicating membranous expression. PLAP IHC. Bar, 20 μm. (**c**) Canine seminoma (same as in Figure 1a). There is strong cytoplasmic staining for c-KIT (arrows). c-KIT IHC. Bar, 20 μm. (**d**) Human classical seminoma. Most cells show strong membranous and some cytoplasmic signal for c-KIT (arrows). c-KIT IHC. Bar, 20 μm.

*PLAP:* Two of 45 sections contained scattered, focal areas with accumulations of strongly PLAP positive tumour cells (Figure 2a). The two positive sections were intratubular seminomas, and both had some PAS positive granules in the tumour cell cytoplasm (see above). All sections in the present study contained peritubular myoid cells and perivascular smooth muscle cells with sparse to strong positive PLAP signal (internal positive controls). The positive control section (human SE) contained areas of varying size with strong positive PLAP signal (Figure 2b). The positive tumour cells formed a clearly visible, focally confluent network, which indicated a membranous staining. In addition, positive cytoplasmic signal was seen in individual tumour cells. No positive cells were seen in the negative control (human SE with primary antibody substituted with an isotype matched irrelevant antibody, mouse IgG-kappa).

*c-KIT:* 32 of 44 sections showed positive staining for c-KIT (Figure 2c), with 12 of 44 sections having no signal. The signal in the positive sections varied considerably, both in distribution and strength (summarised in Table 1). Eighteen tumours had a staining area of less than 25% of the tumour tissue, five stained 25-50%, seven stained 50-75%, while two had a staining area of more than 75% of the tumour tissue. On a semiquantitative scale of staining strength the sections varied from weak (11), moderate (5), strong (8) to very strong (8). The staining pattern also varied, with 19 tumours showing both membranous and cytoplasmic staining, while 13 showed cytoplasmic staining only. One of the two sections with PAS and PLAP positive, intratubular seminoma showed strong membranous and cytoplasmic c-KIT staining in the same cells, while the other showed only scattered tumour cells with cytoplasmic c-KIT staining. The positive control (human SE) stained with a very strong signal in more than 75% of the tumour tissue (Figure 2d). The negative control (human SE with primary antibody substituted with BSA/TBS buffer) was blank.

**Table 1:** **The number of c-KIT positive cases**^**a **^**by area of staining, signal strength and staining pattern based on c-KIT immunohistochemistry in 44 canine seminomas from the Norwegian Canine Cancer Register**

**Staining area**	**#**	**Signal strength**				**Staining pattern**^**b**^	
		**Weak**	**Moderate**	**Strong**	**Very strong**	**MC**	**C**
>75%	2	0	0	0	2	2	0
50-75%	7	0	1	4	2	5	2
25-50%	5	0	2	2	1	4	1
<25%	18	11	2	2	3	8	10
c-KIT positive	32	11	5	8	8	19	13

^a^12 cases were c-KIT negative and are not included in the table.

^b^Staining pattern. M: Membranous, C: Cytoplasmic.

## Discussion

Several studies have recently been performed to determine the histological nature of canine seminomas and to evaluate the feasibility of using this common tumour in dogs as a model for human seminomas [16,18-20]. Three of these studies classified seminomas in the dog as classical (SE) and spermatocytic (SS) seminoma by means of morphological evaluation, PAS reaction and PLAP and c-KIT expression. SE was found in 22/43 cases [16,18] and 8/23 cases [19]. However, Bush et al. (2011) investigated 347 canine seminomas and determined that these were consistent with human SS based upon histological characterization and immunohistochemical analysis (PLAP, c-KIT, DAZ and DMRT-1).

In the present study, none of the 45 examined seminomas were found to be morphologically consistent with human SE. Furthermore, no intratubular germ cell neoplasia unclassified (ITGCNU)/*carcinoma* in situ (CIS)-like patterns of neoplastic cells were detected. These are believed to be the early, pre-invasive stages of SE in man and are commonly seen adjacent to fully developed SE [1]. Two of the cases with intratubular seminoma showed scattered clusters of strongly PLAP positive tumour cells. In both of these cases the tumour cells contained PAS positive cytoplasmic granules and in one case, the PLAP+/PAS + tumour cells also stained strongly for c-KIT. However, this tumour tissue had typical SS morphology. In the other case, only a few of the PLAP + tumour cells were c-KIT positive, and this tissue was also morphologically consistent with SS. According to Ulbright et al. (1999) some SSs may show scattered clusters of PLAP + cells and scant PAS + staining. Thus, due to lack of SE-morphology in these two cases, it was concluded that they were in fact analogous to SS with an intratubular growth pattern.

The distinction between SE and SS is important because the biological behaviour of the two seminoma types are markedly different, resulting in differing treatment strategies and prognostic evaluations [7]. Based on the observed differences in molecular biology and pathological behaviour, the notion was established that the two tumour types originate from two distinct stages in the development of the male germinal cells: SE from the fetal gonocyte stage and SS from the post-pubertal spermatogonial/spermatocytic stage [6].

SE, including its precursor stage, ITGCNU/CIS, is by far the most common type of the two in man, and consequently, the cellular origin of SE has been thoroughly investigated [21-24]. One of the first molecular markers found for SE was PLAP. It has been firmly established that PLAP is positive in very early differentiation stages of male germinal cells, showing strong signals in primordial germ cells, gonocytes and prespermatogonia [22]. These cells are found in fetal testes, and the reliably strong staining for PLAP in ITGCNU/CIS and SE tumour cells of young adult men has led to the widely accepted notion that these tumours originate from the above mentioned cell stages during fetal and/or perinatal life [23]. The early time point for development is underpinned by the observation that most germinal cells in the first trimester testes stain positive for PLAP, while positive cells were rare in the second trimester [25]. Consistently, post-pubertal stages of spermatogonia are PLAP negative [1].

The c-KIT marker has also been identified as a marker for ITGCNU/CIS and SE [8]. This protein, a transmembrane tyrosine kinase receptor, and its ligand, stem cell factor (SCF), have an important function in directing migration and stimulating the survival of male germinal cells, from the primordial stage to the post-pubertal spermatogonial stages [26]. Consequently, the c-KIT protein has been shown to be expressed beyond the point in germinal cell differentiation at which PLAP expression is downgraded. Both mice and rats express c-KIT throughout the post-pubertal stages of A-spermatogonia [26,27].

The extensive staining of c-KIT in our material is not in keeping with the well-established role of this protein as a marker for SE. 32 of 44 sections (73%) showed positive staining for c-KIT, 14 (32%) of which had a staining area of 25% or more, the staining signal varying from moderate to very strong. This is not unlike the findings of Bush et al. (2011), where 40% of the investigated sections were positive for c-KIT staining of the tumour cells in neoplasms that were morphologically, and otherwise, interpreted as SS [20]. In the study of Bush et al. (2011), DMRT-1, a protein important in male development and sex determination, was used to identify SS cells. Studies in mice have shown that DMRT-1 is strongly expressed at the A spermatogonium stage and then downgraded at the B spermatogonium stage, regulating the entry of spermatocytes into meiotic cell division [28]. Human and canine SSs are strongly DMRT-1 positive, indicating that this tumour type originates from pre-spermatocytic stages of germinal cells [29]. Lim et al. (2011) recently showed that human SS might have a more heterogeneous histogenesis than previously anticipated. Their study shows that SS-cells may originate from several stages of post-pubertal spermatogonia, mainly from the late A_pale_ spermatogonium stages and B spermatogonium stages, but also, in a minority of cases from the earlier A_dark_ spermatogonium stage [30]. This suggests that some of the stages of germinal cells that could be the origin of SS may also express c-KIT. In several studies of human SS, relatively high proportions of the tumours were c-KIT positive, leading the authors in one study to propose that at least some SS cases may develop from fetal germ cell stages [31], while others indicated differences in specificity between antibodies used in the immunohistochemical protocol [32]. In a study of 20 canine seminomas, 100% (20/20) were found to be strongly positive for c-KIT, while 20% (4/20) were PLAP positive [33]. Taken together all these results paint a somewhat diverse picture of the expression of c-KIT in germinal tumours. Our interpretation of the c-KIT staining in the present study is that at least some cases of SS may originate from male germinal cell stages in which the expression of the c-KIT protein has not been downgraded.

The lack of SE cases in our material is in contrast with some similar international studies [16,18,19], but in agreement with the traditionally held opinion that canine seminomas are predominantly of the spermatocytic type [17,20]. There might be several factors contributing to this discrepancy. Variability in the occurrence of testicular germ cell tumours among breeds has been described, [14,34,35] and breed or genetic factors could also play a role in the development of SE. The material in the different studies is collected from submissions to diagnostic laboratories and the breed-distribution might not be comparable across studies due to differences in breed popularity between countries. Furthermore, geographical differences could lead to different exposure to environmental factors, which might also contribute to the variability in results between studies. Finally, currently there are no standardised criteria regarding interpretation of PLAP-, c-KIT- and PAS-sections of canine seminomas. Subjective interpretation could possibly have biased the results and contributed to apparent differences between studies. Differences in the specificity of the antibodies used in the different studies could also affect the results, and have been suggested in studies of human SS [32].

The complicating factor of cross-species variation must also be considered. Several characteristics commonly recognised in human SS and SE are apparently not applicable to their canine counterparts. In the current study, multifocal lymphocyte infiltration was recognised in 35/45 (78%) of the diagnosed SSs. In man, this is a common feature of SE, but not in SS [1]. c-KIT positive tumour cells were revealed in 32/44 (73%) examined sections, which is in discrepancy with the role of this protein as a marker for human SE. Both these findings are in accordance with the study of Bush et al. (2011). The mean age for all dogs in our study was 10.1 years. The older age of affected dogs is consistent with the human clinical presentation of SS in the 6^th^ decade. SE, on the other hand, occurs in younger men. However, the age at diagnosis of SE in the study of Grieco et al. (2007) and Kim et al. (2010) were 10.8 years and 8.4 years, respectively. If there are indeed two types of seminomas in dogs, both appear to develop in older subjects. Furthermore, the malignant nature of human SE is not frequently observed among canine seminomas, which are generally characterised by low malignancy and metastatic potential. On the basis of the incongruity in characteristics recognised in human and canine seminomas, one might consider the possibility of canine seminomas originating from other stages of development than their human counterparts.

## Conclusions

The value of canine seminoma as a model for SE in man remains unclear. Among the 45 investigated tumours from Norwegian dogs, none were classified as classical seminoma based on morphological criteria consistent with human seminomas. Regional or breed differences in the occurrence of classical seminoma in the dog, as well as the lack of uniform diagnostic criteria, might explain the discrepancy between the findings in the current study and the results presented by other authors.

## Abbreviations

ABC: Avidin-biotin-peroxidase complex; AEC: 3-amino-9-ethylcarbazole; BSA: Bovine serum albumin; C: Cytoplasmic staining; CIS: Carcinoma in situ; DAZ: Deleted in azoospermia; DMRT-1: Doublesex and mab-3 related transcription factor 1; EDTA: Ethylenediaminetetraacetic acid; HE: Hematoxylin and eosin; HPF: High power field; HRP: Horseradish peroxidase; ITGCNU: Intratubular germ cell neoplasia unclassified; MC: Membranous and cytoplasmic staining; PAS: Periodic acid-Schiff; PBS: Phosphate buffered saline; PLAP: Placental alkaline phosphatase; SCF: Stem cell factor; SE: Classical seminoma; SS: Spermatocytic seminoma; TBS: Tris buffered saline; WHO: World health organization.

## Competing interests

The authors declare that they have no competing interests.

## Authors’ contributions

TET drafted the manuscript with contributions from AN, TG and GG. GG carried out the routine histology and immunohistochemistry with assistance from TET. TET, AN, TG and GG in collaboration, conceived of and participated in the design of the study. All authors read and approved the final manuscript.
